# Glycosaminoglycans in Tissue Engineering: A Review

**DOI:** 10.3390/biom11010029

**Published:** 2020-12-29

**Authors:** Harkanwalpreet Sodhi, Alyssa Panitch

**Affiliations:** 1 Department of Biomedical Engineering, University of California Davis, Davis, CA 95616, USA; hssodhi@ucdavis.edu; 2 Department of Surgery, University of California Davis, Sacramento, CA 95817, USA

**Keywords:** glycosaminoglycans, tissue engineering, extracellular matrix, chondroitin sulfate, hyaluronic acid, dermatan sulfate, keratan sulfate, heparan sulfate

## Abstract

Glycosaminoglycans are native components of the extracellular matrix that drive cell behavior and control the microenvironment surrounding cells, making them promising therapeutic targets for a myriad of diseases. Recent studies have shown that recapitulation of cell interactions with the extracellular matrix are key in tissue engineering, where the aim is to mimic and regenerate endogenous tissues. Because of this, incorporation of glycosaminoglycans to drive stem cell fate and promote cell proliferation in engineered tissues has gained increasing attention. This review summarizes the role glycosaminoglycans can play in tissue engineering and the recent advances in their use in these constructs. We also evaluate the general trend of research in this niche and provide insight into its future directions.

## 1. Introduction

**Figure 1:**
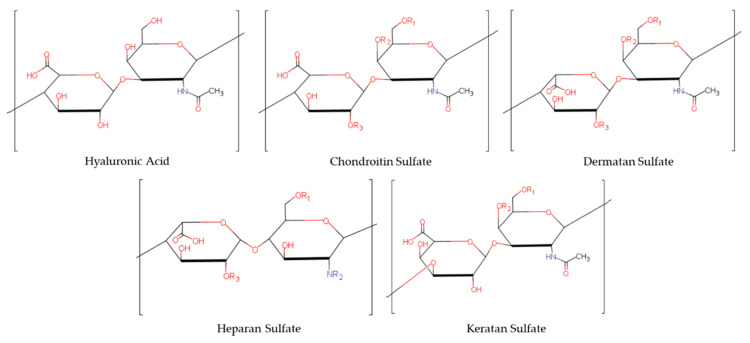
Repeating disaccharide unit of each glycosaminoglycan. “R” indicates a potential sulfation point.

Glycosaminoglycans (GAGs) are long, unbranched polysaccharide chains made up primarily of repeating disaccharide units. These disaccharide subunits are composed of one hexuronic acid and one amino sugar linked by glycosidic bonds [1] and these variations in disaccharide composition are used to distinguish the major classes of GAGs: Hyaluronic Acid (HA), Chondroitin Sulfate (CS), Dermatan Sulfate (DS), Keratan Sulfate (KS), and Heparan Sulfate (HS). GAGs are sulfated to varying degrees, with the exception of Hyaluronic Acid (HA), which is unsulfated. The different hexuronic acids and amino sugars found in each GAG are summarized in Table 1 and a structural diagram of the repeating disaccharide unit of each GAG is provided in Figure 1. CS, DS, and HS range in molecular mass between 10,000 and 50,000 Daltons and KS and Heparin (a GAG similar to but distinguished from HS) range between 5000 and 15,000 Daltons. In contrast, HA is generally a very high molecular weight GAG, ranging between approximately 100,000 and 10,000,000 Daltons [2]. The presence of the ionizable groups (sulfates and carboxylates on hexuronic acids) confers GAGs with polyionic properties that are responsible for their key abilities such as water retention, cell binding, control of ion fluxes and neuronal signaling [3,4,5].

The first reference to GAGs can be found in electronically available published literature dating back to the late 1930s when Karl Meyer summarized GAG chemical properties and biological relevance known at the time. Then, they were referred to as mucopolysaccharides and were classified primary as “containing iduronic acid”, with sub-divisions of sulfate-free and sulfate-containing, or “neutral” [6]. Even at this time, knowledge of their general localization within the body was growing. It was known, for example, that “chondroitinsulfuric acid” could be isolated from cartilage, the aorta, and the sclera and also that its presence was decreased in “rachitic” (weak) bones [6]. Fast forward to the 1950s and researchers had identified that cell excretion of mucopolysaccharides could be used to determine the differentiation of fibroblasts in culture [7]. Research in this time period focused on isolating and characterizing new GAGs and elucidating the expression patterns and purpose of GAGs in the body [8], during development [9] and disease [10,11,12]. Foreshadowing the discovery of the importance of GAGs in tissue remodeling and applications in tissue engineering, in 1958, Bollet et al. analyzed the GAG content of granulation tissue formed when polyvinyl sponges were implanted under the dorsal skin of guinea pigs [13].

**Figure 2:**
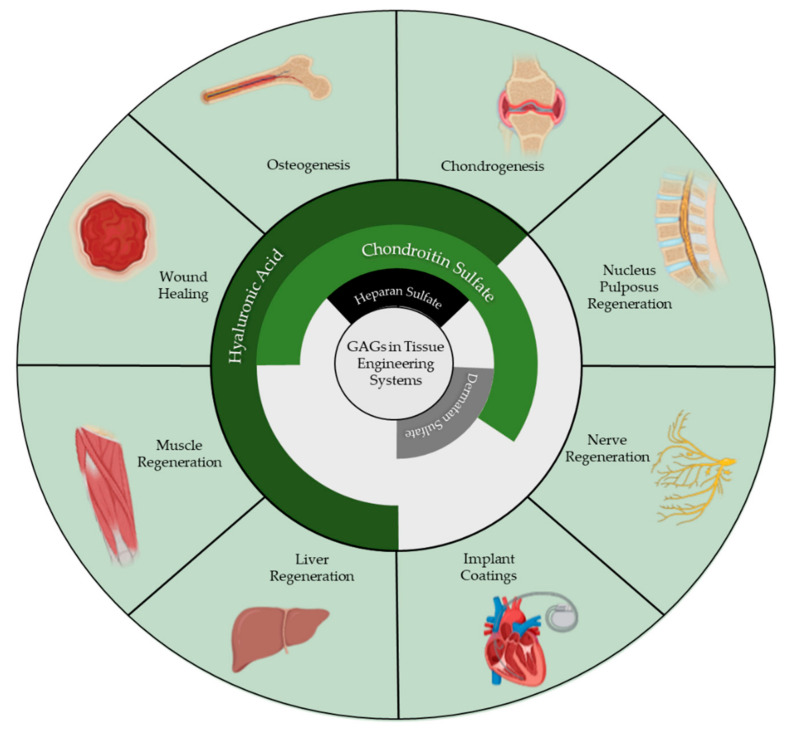
Each GAG and their aforementioned tissue engineering applications. This figure was made using BioRender.

The first research using GAGs in tissue engineering scaffolds arose in the 1980s, with scientists investigating hyaluronic acid as a component of scaffolds for regeneration of tissues. Since then, all GAGs, with the exception of Keratan sulfate, have seen increased utilization in tissue engineering constructs for the treatment of a myriad of diseases such as osteoarthritis, neuropathy, and bone defects, to name a few. This review aims to summarize the use of each GAG in the advancement of tissue engineering in the last five years, depicted pictorially in Figure 2, and project how, as GAGs become more thoroughly understood, their utility and ubiquity in the tissue engineering field will expand.

## 2. Hyaluronic Acid

HA, also referred to as hyaluronan, has a repeating disaccharide unit of D-glucuronic acid and N-acetyl-D-glucosamine (the hexuronic acid and amino sugar, respectively) attached by a beta 1–3 bond, and the disaccharide units are joined by a beta 1–4 bond [1]. [1] It is found in liquid connective tissues such as the synovial fluid of joints and the vitreous humor of the eye where, in conjunction with other charged constituents of the extracellular matrix, it plays a key role in maintaining viscoelasticity via water retention due to both hydrogen bonds and osmotic pressure generated from the high density of anionic groups and accumulation of counter cations. When in water, HA has a gelatin-like consistency. Its viscoelasticity and ability to form matrices that retain water allow it to cushion joints, resist compression and help reduce friction in all joint tissues [3]. It also plays a role in the extracellular matrix of several tissues where it mediates receptor-driven detachment, mitosis, and migration. This control of cell division and cell migration means HA is commonly implicated in tumor development and cancer metastasis [3]. HA is the highest molecular weight GAG [1], presenting with a broad range of molecular masses generally ranging between 10^5^ and 10^7^ Daltons. This is in stark contrast to other GAGs, which are generally on the order of 103 Daltons [2]. HA’s molecular mass plays an important role in its function. Studies have shown that HA fragments of varying lengths may alert the body to trauma and play roles in the progression of wound healing. Degradation of HA increases tissue permeability and HA fragments enhance angiogenesis, promoting tissue healing processes [3]. In contrast, endogenous HA has been shown to promote extracellular matrix secretion, reduce inflammation, and inhibit immune cell migration to maintain homeostasis in healthy tissue [3].

HA has many properties that make it an ideal candidate for tissue engineering scaffolds. It is biodegradable, biocompatible, and resorbable. HA is involved in every step of wound healing in the body [14]. The interplay between its hydrophilicity and control of cell migration allows HA to form a temporary, ideal wound healing environment. Because HA is hygroscopic, it can control the hydration of tissue during healing, allowing for increased flow of nutrients and effluence of waste products [15]. It also stimulates cells via interactions with CD44, RHAMM, and ICAM-1 cell receptors, which allows it to regulate cell adhesion, motility, inflammation, and differentiation [14]. Despite this, for most cell types it does not support sufficient attachment or spreading and requires chemical modification to support cell growth and survival [16].

In its native form, HA is a weak scaffolding material because it is rapidly degraded in vivo by hyaluronidase and is highly soluble, which can cause dissolution. It must, therefore, be chemically modified and crosslinked or combined with another polymer to form stable, structurally integrated scaffolds that support cell adhesion and proliferation [17]. Encouragingly, HA can be crosslinked under basic, acidic, and neutral pH conditions or combined with other natural and synthetic polymers to confer strength, allowing for diverse applications such as treating difficult to heal wounds, burns, and any form of trauma that requires a space-filling scaffold [14].

Additional support for chemical modification of HA comes from the fact that HA itself does not bond to surrounding tissue when it is used to fill defects, and it is often of sufficiently high molecular weight that it does not diffuse into the surrounding tissue to form an integrated seal when crosslinking it in situ. In addition, while viscous HA gels can be injected, injection of unmodified HA has been shown to cause damage and hemorrhaging in some tissues, such as in the heart and liver. With the goal of overcoming all of these shortcomings, Shin, et al. developed a tissue adhesive HA hydrogel functionalized with the adhesive catecholamine motif from mussel foot protein. This gel was shown to reduce apoptosis, increase viability, and enhance the function of human adipose-derived stem cells and hepatocytes. HA-catecholamine laden with hepatocytes was shown to gel and adhere to the liver of athymic mice within minutes. Further, the gel was still present after two weeks and albumin secreted by the transplanted hepatocytes was detectable in the blood stream 3 days after implantation [18], indicating some recapitulation of endogenous tissue behavior.

### 2.1. Hyaluronic Acid Supports Multiple Crosslinking Mechanisms

While not always supportive of in situ bonding with the surrounding tissue, crosslinking HA is an important tool and can affect the way in which cells interact with HA. While non-crosslinked or loosely crosslinked HA scaffolds do not support the growth of human mesenchymal stem cells (MSCs) due to their low strength and non-adhesive properties, Lou, et al. have developed a viscoelastic HA hydrogel capable of supporting cell adhesion and spreading [19,20]. HA-hydrazine can form transient, non-covalent, hydrazone bonds with HA-aldehyde and/or HA-benzaldehyde. Aldehyde functionalized HA was found to have an order of magnitude faster dissociation and association rate with HA-hydrazine than HA-benzaldehyde. Therefore, increased concentrations of HA-aldehyde results in slower stress-relaxing gels, and increased HA-benzaldehyde results in faster stress-relaxing gels [19]. A hydrogel system composed of both allowed control over gelation time and stress relaxation time. Stress relaxation time, as measured by 50% relaxation of initial stress, was tuned broadly from as slow as 5 h to 20 min by switching from HA-benzaldehyde to HA-aldehyde. Gel storage modulus was also varied from as low as 8.2 ± 1.1 Pa up to 471 ± 31.2 Pa by varying the molecular weight of HA and total HA concentration [19]. These scaffolds have been seeded with human MSCs [19] and human adipose derived stem cells [20] and shown to be cytocompatible and support cell spreading at certain ratios of HA-aldehyde and HA-benzaldehyde. This system is highly adaptable and can also be augmented with other biomolecules. The addition of collagen added cell-binding motifs not present on HA, affected viscoelasticity, and added a fibrillar component to the gels [19]. The addition of cellulose nanocrystals increased network organization and stiffness while also increasing degradation time [21]. The addition of neither collagen nor cellulose nanocrystals adversely affected the ability of HA gels to support cell growth [19,20,21].

In addition to modulating the viscoelastic properties through creation of transient crosslinks, several techniques have been developed to covalently crosslink HA through reaction of the carboxylate groups on the GAG backbone. In applications related to musculoskeletal tissue engineering, HA based polymer systems have also been shown to trigger endochondral bone formation in vitro and in vivo [22], making it a prime candidate for bone tissue engineering constructs. Poldervaart, et al. have shown that, not only can methacrylated HA support osteogenic differentiation, it can also be 3D printed [23]. 3D bioprinting allows for creation of porous scaffolds with a predefined shape and incorporation of cells and signaling molecules within the constructs in predetermined locations [24]. These photocrosslinkable gels showed long-term stability, lasting up to 14 days in the presence of hyaluronidase at 3% gelatin versus 1–7 days at lower gelatin concentrations. They also exhibited high stiffness, with storage moduli ranging from 170 ± 63 Pa up to as high as 2602 ± 199 Pa and elastic moduli as high as 10.6 kPa depending on concentration of HA (1–3% *w*/*v*) [23]. When seeded with bone marrow-derived human MSCs, cell viability at 21 days remained at 64.4% and MSCs showed spontaneous osteogenic differentiation without additional stimuli. While not required, bone morphogenic protein 2 accelerated mineral deposition within these constructs [23]. Similarly, gels made of esterified HA have also been shown to support osteogenic differentiation when seeded with bone marrow concentrate [25]. Bone marrow concentrate allowed MSCs to remain surrounded by their native microenvironment and circumvented the difficult process of pure MSC extraction, while esterified HA provides mechanical support [26,27]. Thus, HA is compatible to various crosslinking modalities.

Current research also explores new hydrogel manufacturing methods that not only overcome the intrinsic weaknesses of HA, but also avoid the use of organic solvents/reagents that cause toxicity and also allow for new types of HA constructs. HA microporous gel systems, where microgels are combined with cells and other additives and then crosslinked to form a macrogel, have been developed to overcome not only nano-porosity issues common with other gels, but also to allow for non-toxic crosslinking after cell seeding [28]. Microporous gels have enhanced cell and tissue integration properties when compared to nano-porous gels [28]. These newer microporous gels improve upon first generation gels that used techniques such as salt leeching [29], gas foaming [30], or using harsh chemicals for creating micropores in gels. Using these older techniques, cells and signaling factors needed to be seeded after pore formation and cell infiltration was limited and slow. Using the newer microbead technique, acrylamide-functionalized HA was formed into crosslinked microspheres using microfluidic droplet generation. The microgels were crosslinked using dithiol matrix metalloprotease sensitive linker peptides. Subsequently, microgels were mixed with human dermal fibroblasts and microbeads crosslinked to one another using light-induced free radical polymerization or carbodiimide chemistry to form a bulk microporous gel. This system allowed for the rapid cell infiltration necessary for endogenous tissue integration without significant scaffold degradation often seen in other hydrogel technologies that use cell remodeling of the matrix to allow for cells to enter [28]. This allows the gel to remain implanted longer while retaining its original mechanical properties, which is important for enhancing cell lineage specification and retention [31].

### 2.2. Hyaluronic Acid Blends

More control over gel properties and better recapitulation of the extracellular matrix are possible by combining HA with other natural polymers that confer mechanical strength, cell binding motifs, and change the microstructure of the gel. Gelatin is an ideal copolymer for HA as it provides structural support and RGD-integrin binding sites that allow cell adhesion and proliferation, unlike HA alone [32]. Gelatin-HA constructs have been studied extensively for regeneration of articular cartilage [33,34,35], wound healing [36,37,38], and even vocal fold repair [39] due to their chondrogenic, angiogenic, and cell adhesive properties, and their tunable viscoelastic properties. Like HA, gelatin can be methacrylated, which allows for photopolymerization of gelatin-gelatin or gelatin-HA crosslinks. Constructs made of methacrylated gelatin and HA have been shown to suppress hypertrophy and increase GAG expression by embedded, human bone marrow stem cells and, when tested in a full thickness osteochondral defect in rabbits, showed good cartilage regeneration [33]. Methacrylated HA and methacrylated gelatin can also be 3D bio-printed and polymerized with embedded cells without affecting their viability or chondrogenic properties, making them a good platform for custom, patient-specific cartilage implants [35]. Feng, et al. have also shown that a slightly different chemistry involving thiolated gelatin and HA-vinylsulphone can form hybrid microgels, generated from crosslinked microbeads, similar to the HA-only microgels mentioned previously. Human bone marrow stem cells encapsulated in HA-gelatin microgels showed high viability and chondrogenic potential. When injected subcutaneously in mice, the cell-laden gels formed smooth, elastic, cartilage-like tissue, and reduced hypertrophy and vascularization over the course of 8 weeks [34].

This ability to drive cell behavior is also augmented by the slow degradation rate of gelatin. While HA alone promotes angiogenesis, decreasing gel degradation rate and providing cell binding sites, via complexation with gelatin accelerates healing and decreases counterproductive inflammatory cell migration at wound sites [36]. When carbohydrazide gelatin was combined with HA-monoaldehyde, they formed an injectable gel that showed no toxicity when tested with human umbilical cord endothelial cells in vitro [36]. Further, gels tested using an ex vivo rat aortic ring assay showed endothelial invasion and microvascular extension into the gel at the aortic ring-gel interface, supporting the hypothesis that HA, which can be angiogenic on its own at the correct concentrations [36,40], can be enhanced with a polymer that presents cell binding sites and slows gel degradation.

In wound healing, where angiogenesis is critical, Ebrahimi, et al. showed that electrospun gelatin-HA constructs could accelerate healing of thermal burns in mice [41]. In contrast to most other natural polymers, gelatin constructs can also be electrospun to generate nanofibrous gels instead of standard monolithic ones. Nanofibrous scaffolds structurally mimic the fibrillar structure of the extracellular matrix, allow for cell adhesion due to the high surface area to volume ratio, allow oxygen to permeate, and allow cell waste to escape, all while inhibiting pathogen infiltration [42], making them excellent candidates for wound healing applications. Similar electrospun constructs have been made using gelatin and HA combined with chitosan, which showed success in a mouse model of wound healing [43] and rabbit models of alkali induced corneal burns [44]. All of these constructs reduced inflammation and improved healing, demonstrating the potential improvement of gelatin-HA construct using a nanofibrous structure. However, nearly all of them, with the exception of acetic acid-based gel systems, use harsh solvents for electrospinning and crosslinking, making their use cumbersome and potentially hazardous. In situations where complex functionalization of the scaffold is required, an easily modifiable polymer such as poly(caprolactone) [45] can be used to electrospin HA instead of gelatin. Poly(caprolactone) (PCL) has been used extensively in biomaterials, especially for electrospinning, but it lacks the cell signaling characteristics and hydrophilicity of HA. PCL electrospun scaffolds doped with HA and epithelial growth factor have been shown to promote cell infiltration while also up-regulating collagen and TGF-ß1 expression in vitro. In vivo, the HA-PCL gels, when doped with endogenous growth factors, showed regeneration of a thicker epidermis layer and formation of an organized dermis layer as well in a rat model of full thickness skin wound healing [42]. Like the HA-Gelatin electrospun constructs, this system also employs harsh solvents, such as chloroform, leaving room for improvement in the electrospinning of nanofibrous HA scaffolds [42].

Less commonly, HA-gelatin solutions have been investigated for regeneration of muscle tissue and as a model system for lung tissue. Gelatin and HA can both be functionalized with tyramine to allow for gelation using horseradish peroxidase and H2O2 [32,46]. C2C12 murine myoblasts seeded on these tyramine crosslinked scaffolds were shown to retain myoblast differentiation and myotube formation, while HA-only and Gelatin-only gels did not. HA gels supported spherical cell morphology due to lack of cell binding sites in HA, and gelatin gels showed dedifferentiation, as the gel collapsed under cell traction forces [32]. Kumar, et al. also showed that tyramine-functionalized HA and gelatin could be spin-coated into membranes and seeded with cells to generate an in vitro model of the alveolar basal epithelium for lung-based research. The films supported attachment, migration, and proliferation of alveolar basal epithelial cell line A549. When laden with growth factors, the membranes also induced some epithelial differentiation in MSCs [37]. Taken together, this research is suggestive of the vast potential of HA blended with gelatin and other bioactive species for tissue regeneration. It also highlights the array of crosslinking and manufacturing modalities that are under investigation to produce fully functional HA-based tissue engineered constructs.

While HA-gelatin blends are promising materials, many other HA blends have been investigated and have also shown promise. Tyramine functionalization of HA has been studied in combination with silk polymers for tissue engineering constructs. Raia, et al. have shown that HA-tyramine and silk fibroin-tyramine can be covalently crosslinked to form tunable hydrogels that begin to approach relevant mechanical properties and overcome some of the inherent weaknesses of HA [47]. In this study, silk fibers formed di-tyrosine bonds via horseradish peroxidase, resulting in highly elastic gels containing crystalline regions of silk. Tyramine-substituted HA, on the other hand, formed weak hydrogels that degraded rapidly. Use of a combination of both biopolymers overcame these weaknesses and resulted in tunable scaffolds. HA concentration in the matrix allowed adjustment of gelation time, degradation rate, and water retention. HA only hydrogels degraded within 6 days, while silk gels retained 70% of their mass on day 6. Hybrid gels allowed for tuning rate of degradation within this range of 1–6 days [47]. Silk-HA gels also achieved 100% strain before breaking, versus 30% in HA-only gels. Silk-only and HA-only gels exhibited storage moduli of 2.27 ± 0.09 KPa and 0.55 ± 0.03 KPa, respectively, while hybrid gels achieved moduli slightly beyond this range, peaking at 3.85 ± 0.08 KPa [47]. Silk gels alone were shown to allow adhesion and promote proliferation of human MSCs and this property was conferred to silk-HA hybrid gels. HA-only hydrogels inhibited MSC growth, showing an unadhered, spherical morphology after one week [47]. Combining silk and HA in this gel construct augments HA with the mechanical strength and degradation properties necessary to support cell growth with fine control over gel mechanical properties.

Similarly, tunable hybrid gels have been developed using HA and agarose. In contrast to HA, agarose has good gelatinizing properties, but exhibits slow degradation, limiting its use in some tissue engineering applications, which often target replacement of the engineered scaffold with host tissue [48]. Chu, et al. have shown that grafting of HA to agarose activated with epichlorohydrin resulted in a scaffold that presented the same cell regulation motifs as HA alone but also supported cell adhesion and proliferation. The gels were shown to stimulate TNF-α secretion in RAW 264 macrophages and upregulate Collagen I and III secretion by 3T3 fibroblasts. Further, when tested in a murine model of full thickness wound healing, agarose-HA gels showed rapid healing when compared to controls over the course of 21 days, showing that HA can facilitate wound healing past 1 week when combined with a slowly-degrading polymer [49].

While this list of HA-polymer blends is not exhaustive, it does demonstrate the enormous potential and versatilely of HA. HA interacts with cell receptors that regulate inflammation, cell differentiation, and cell motility, making it useful for a myriad of tissue engineering applications. However, it forms weak gels alone and does not adhere to tissues or support cell adhesion through integrin receptors thought to be required for tissue regeneration. These weaknesses can be overcome by functionalizing and crosslinking HA or combining it with another polymer, such as silk fibroin, gelatin, collagen, agarose, or polycaprolactone, which can provide strength and cell binding sites. In an appropriate scaffold, HA has been shown to induce chondrogenesis, osteogenesis, and wound healing by driving stem cell behavior. The ubiquitous nature of HA within the body and the ease with which it can be functionalized and combined with other polymers fully supports continued exploration of HA for successful development of tissue engineered products. A summary of HA tissue engineering constructs and their tested behavior can be found in Table 2.

## 3. Chondroitin Sulfate

CS is composed of a repeating disaccharide made up of D-Glucuronic acid, a hexuronic acid, and N-acetyl-d-galactosamine, an amino sugar. It is generally highly sulfated with -SO3 occuring at C4 or C6 on galactosamine [1]. Four subsets for CS exist: A, C, D, E. These subsets are differentiated by the location of the sulfates in the sugar rings. CS type B has subsequently been classified as dermatan sulfate; another GAG discussed later [50]. CS is an integral part of solid connective tissues such as cartilage, bone, skin, ligaments, and tendons [51]. Similar to HA, CS, when bound to a proteoglycan such as aggrecan, plays a key role in retention of water, due to the high density of anionic groups, and resistance to compression making it key in the cushioning and lubrication of joints [50,51].

Chondroitin sulfate-based gel systems have been developed for cartilage [52] and other tissue repair [53]. Similar to HA, chondroitin sulfate has the capacity to induce cell differentiation, making it useful in chondrogenic and osteogenic constructs, however, unmodified and alone, it also lacks the essential mechanical properties necessary for implantation into tissues [54] including cartilage, bone defects, or the nucleus pulposus (NP). Unlike HA, CS promotes cell adhesion and can be used to make non-adhesive polymers adhesive to cells [54]. The bulk of current research, therefore, focuses on adding CS moieties to tissue engineering constructs while mimicking the physical properties of native tissue. This can be done by incorporation of free CS chains into a different bulk material, crosslinking CS to itself or to another polymer [55,56,57,58]. In most systems, CS is conjugated with a covalent crosslinker that allows for self-gelation or gelation into a multicomponent matrix. In some systems, CS is entrapped in a matrix and allowed to diffuse in a manner controlled by mesh size and charge interactions [59]. The exact effects of immobilization technique on cell response to CS is still not well understood. However, the wealth of studies incorporating CS is shedding light onto biological activity inherent to CS.

### 3.1. Sulfation and Sulfation Pattern Suport Biological Activity

In bone tissue engineering, CS is responsible for coordinating osteoblast attachment, cell lineage commitments, and differentiation [60,61]. CS also interacts with growth factors critical for bone regeneration [61]. As such, CS scaffolds have the potential to replace the collagen scaffolds impregnated with Bone Morphogenic Protein 2 (BMP-2) that are currently the medical gold standard for treating critically sized bone defects. BMP-2 is an osteoinductive growth factor. The human recombinant form is approved by the FDA and used clinically with collagen sponges when autograft and allograft are not feasible to repair a bone defect. Andrews, et al. demonstrated extended release of recombinant human BMP-2 from CS scaffolds compared to their collagen sponge counterparts. CS based scaffolds showed very similar total release as compared to collagen gels after 15 days. However, the time to 50% BMP-2 release was 1.5 days for collagen, versus 5 days for CS gels, demonstrating a much more linear release profile, despite comparable total release [62]. Unmodified CS alone cannot, however, form structurally integral scaffolds that support cell growth and be implanted in the body. A common way to overcome this limitation is to methacrylate CS, which allows the polysaccharide chains to be crosslinked via photopolymerization using UV light and a photoinitiator such as 2-hydroxy-4′-(2-hydroxyethoxy)-2-methylpropiophenone [62]. When used to treat a challenging critically sized femoral defect in rats, methacrylated CS scaffolds loaded with BMP-2 induced comparable bone formation to the BMP-2 in collagen sponges as measured by bone volume, strength, and stiffness [62], despite the improved release kinetics of CS based gels. This could be due to the more rapid release of BMP-2 from collagen gels, which showed an initial burst release and demonstrated a collagen deposition pattern characteristic of more mature bone than CS gels [62]. Taken together, these data show the potential of CS-based systems to improve growth factor release kinetics and induce osteogenesis at a level that, at the very least, is equivalent to the current gold standard [62]. This is due in part to the osteogenic interactions between cell surface receptors and CS, but also due to the ability of CS to sequester and release growth factors in a controlled manner via growth factor interactions with the sulfated CS.

This affinity for growth factors and ability to control growth factor presentation to cells also confers CS with the ability to drive neuronal regeneration [63,64]. Besides osteogenic factors like BMP-2, methacrylated CS scaffolds have a strong affinity for fibroblast growth factor 2 (FGF2) and brain derived neurotrophic factor (BDNF), which can be added directly or via impregnation with platelet-rich plasma that contains these growth factors. This affinity for charged growth factors is strong enough that FGF-2 and BDNF release has been shown to be sustainable for 15 days, significantly longer than release from platelet rich plasma alone [63]. The degree of CS sulfation and sulfation patterns affect growth factor binding and release in addition to other cellular responses. Karumbaiah, et al. investigated the effects of disulfated and monosulfated CS on neurotrophic factor binding, neuronal homeostasis and the influence of variably sulfated CS in biomaterials on neural stem cell fate [65]. They found that binding of neurotrophic factors is dependent on CS sulfation and varies between mono and disulfated CS constructs. In addition, they confirmed the cytocompatibility of methacrylated CS gels for neuronally derived cell lines and demonstrated their ability support self-renewal of rat neurospheres. In other studies, when seeded with embryonic chick dorsal root ganglia, CS gels yielded better nerve growth than their HA counterparts [64]. These studies also showed that control over growth factor binding and direction of nerve growth are dependent on the sulfation patterns [64,65]. Therefore, scaffolds containing CS with the appropriate sulfation patterns can potentially be used in combination with growth factors to encourage and direct nerve growth more effectively than commonly used HA scaffolds. Further, sustained controlled release of growth factors utilizing GAGs such as CS may limit systemic exposure and subsequent unintended physiological responses.

Methacrylation of CS also allows for covalent crosslinking to form scaffolds that not only control growth factor presentation, but also drive bone mineralization. Calcium and phosphate are critical components for bone inorganic structure [66,67,68], so in addition to the need for release of growth factors for bone regeneration, there is a need to support the growth of the ceramic component of these composite tissues. A myriad of methods for incorporating calcium and phosphate ions into biodegradable scaffolds have been explored for bone tissue engineering [69,70,71]. Hydrogels that provide nucleation points for hydroxy apatite, such as ethylene glycol methacrylate phosphate (EGMP), induce faster apatite growth [71]. Kim, et al. have shown the methacrylated CS can be crosslinked to polyethylene glycol diacrylate (PEGDA) to form a gel which promotes nucleation of hydroxy apatite and sequesters the necessary calcium and phosphate ions, thanks to the charged sulfate groups on CS [72]. PEGDA was selected as a bioinert copolymer that is easy to handle, easily seeded with cells [72] and allows for variation of CS concentration in the system to elucidate the relationship between CS concentration and ion sequestration/deposition. Calcium and phosphate ion concentration in the gel was positivity correlated with CS concentration, and PEGDA-CS gels developed white particulate coatings in the presence of phosphate buffered saline [72], indicating PEGDA + CS is able to provide nucleation points for calcium and phosphate deposition. When embedded with human tonsil-derived MSCs, this gel technology demonstrated acceleration bone mineralization relative to controls and showed ion binding and distribution within negatively charged hydrogel was dependent on CS concentration. Furthermore, the biomineralizing microenvironment induced osteogenesis and deposited calcium and phosphate showed a native hydroxyapatite structure. When tested in a mouse model of critically sized calvarial defect, the cell-laden PEG-CS gels showed 2–3 times higher regeneration volume than controls [72]. Miyamoto, et al. have shown a similar ability of CS to induce hard tissue generation when combined with sodium alginate [73]. Together, these studies highlight the multifunctionality of CS, i.e., growth factor binding and nucleating calcium phosphate deposition, and the important role it plays in tissue regeneration.

### 3.2. Chondroitin Sulfate Blends

While methacrylated CS forms mechanically robust hydrogels that retain the inherent functionality of CS and have been shown to support chondrogenesis, osteogenesis and neurogenesis in vitro and in vivo, they require photopolymerization, meaning, in clinical applications, these constructs would require specialized tools for delivery and ultra-violet light for polymerization, which could potentially injure surrounding healthy tissue [74]. Tang, et al. approached this issue by developing a hydrogel scaffold comprised of CS functionalized with graphene oxide. Johnson–Claisen rearrangement chemistry allowed graphene oxide (GO) to be functionalization with primary amines. A solution composed of CS and modified GO gelled in situ within 10 min and the incorporation of graphene improved stiffness and toughness drastically (320 and 70%, respectively) over gels made of just CS. They also proved to be highly porous, resistant to degradation, and enabled MSCs to proliferate and deposit collagen matrix [75]. Of note, however, the entrapment of cells and potential chemical remnants from the EDC/NHS chemistry did initially slow cell metabolism [75]. Potentially of value is the conductive nature of GO, which may confer the scaffolds with the ability to induce natural conductive currents to improve tissue regeneration.

New CS crosslinking methods provide new ways to study the interactions of CS with cells in the absence of other extracellular matrix components and, in that regard, are indispensable. However, CS is not the sole constituent of the extracellular matrix in any tissue in the human body, but rather is interlaced with other GAGs and natural polymers. Therefore, combining CS with different polymers presents an opportunity to further control a gel’s rheological properties, present additional biological signals, and better mimic native tissue. For example, collagen scaffolds functionalized with CS have been shown to recapitulate the chondrogenic niche, modulate inflammation, and mimic the mechanical properties of native collagen [55]. Corradetti, et al. demonstrated that such constructs support chondrogenic differentiation in rat bone marrow-derived stem cells in vitro and suppressed inflammation in vivo. MSCs grown on CS-collagen constructs aligned with scaffold pores, whereas cells grown on scaffolds containing only collagen showed clustering behavior, demonstrating that the presence of CS in the CS-collagen scaffolds is essential to influence cell-scaffold adhesion and [55], therefore, cytoskeletal organization and differentiation [76,77]. These cells also developed more intracellular vesicles, which have been associated with enhanced intercellular communication [78]. The constructs innately induce chondrogenic differentiation, and even though they didn’t support osteogenesis innately, they displayed a synergistic effect with osteogenic media, showing increased expression of osteogenic factors Alp, Spp1, and Bgla compared to controls [55].

CS has also been combined with collagen using genipin as a crosslinker for tissue engineered scaffolds for regeneration of different types of cartilage, such as the nucleus pulposus. Forming a lightly crosslinked, gelatin-like scaffold, type II collagen and CS crosslinked with genipin are biocompatible and support differentiation of adipose-derived stem cells in vitro. When used as an injectable carrier of adipose derived stem cells in a rat model of NP degeneration CS-collagen gels showed increased disc height, water content, proteoglycan and type II collagen synthesis, and partial recovery of NP structure [57].

### 3.3. Processing Techniques and Manufacturing

As tissue engineering systems become more advanced, research naturally trends towards improving their utility in the clinic. Recent studies using CS in tissue engineering, therefore, explore ways to make cell-seeded CS scaffolds injectable and tailorable to individual patients. Injectable tissue engineering constructs are advantageous as they do not require invasive surgeries to implant. Chen, et al., for example, have developed, an enzymatically crosslinked, injectable, and biodegradable hydrogel system comprised of carboxymethyl pullulan and chondroitin sulfate functionalized with tyramine. These conjugates are crosslinkable under physiological conditions using horseradish peroxidase (HRP) and hydrogen peroxide. Porcine articular chondrocytes embedded in these gels demonstrated proliferation and enhanced cartilage-like extracellular matrix deposition over controls, indicating chondrogenesis [79]. This HRP crosslinking method has the potential to form minimally invasive, injectable hydrogels for a myriad of tissue engineering applications, as the molecular weight ratios, polymer concentrations, and crosslinker concentration can all be modified to fine tune gel properties [80,81]. Li, et al. took this one step further and developed a similar system using oxidized CS and pullulan functionalized with adipic hydrazide that is self-gelling and forms in situ. Similarly, this system demonstrated good biocompatibility and chondrogenic properties [82], and supports the concept of developing in situ gelling CS scaffolds for tissue engineering.

A common way to make cell scaffolds injectable is to make them sheer thinning or thermo responsive via combination with a polymer like Chitosan. Chitosan is broadly used for the synthesis of injectable hydrogels due its biocompatibility [83] and thermosensitive capabilities [84]. CS has been combined with chitosan-poly(hydroxybutyrate-co-valerate) in the form of a nanoparticle for nucleus pulposus regeneration. Similar to CS + collagen systems, this hydrogel system supports viability, adhesion, and chondrogenic differentiation of adipose derived stem cells and shows potential for NP regeneration [85]. Alinejad, et al.’s work provides evidence that the gels made with chitosan and CS can be prepared with weak bases such as sodium hydrogen carbonate and beta-glycerophosphate to form thermosensitive, injectable and biocompatible scaffolds with tunable physical properties. Cytocompatibility of these hydrogels scaffolds was also shown to be good. When evaluated with L929 fibroblasts, they showed high viability and metabolic activity for up to 7 days. This effect was enhanced by the addition of CS relative to controls. [56]. CS can also be linked to a chitosan scaffold if the chitosan is functionalized with hydroxy butyl groups and the CS is oxidized, allowing them to crosslink via the Schiff-base reaction [58]. These injectable gels also show good biocompatibility and support adipose derived stem cells, while not eliciting an immune response [58]. This injectable system, however, differs, in that a pre-gel of oxidized CS and hydroxy butyl chitosan can be injected and subsequently completely gelled by injecting more oxidized CS. The authors see this as applicable in molding processes for custom made tissue engineering constructs that are shaped to the patient [58].

Injectable gels also open the door to 3D bioprinting of tailored, patient-specific constructs. Bioprinting generates 3D scaffolds with reproducible and complex structures and offers the opportunity to generate customized hydrogel scaffolds with a predetermined pattern, shape, and size. Engineered cartilage plugs, for example, can potentially be sized to a patient’s joint and shaped exactly to match the defect they aim to repair. In order to 3D print a tissue engineering scaffold, the gel used must have the correct rheological properties to be extruded and undergo rapid gelation upon deposition [86]. Abbadessa, et al. have combined photopolymerizable methacrylated CS with thermosensitive poly(N-(2-hydroxypropyl) methacrylamide-mono/dilactate)-polyethylene glycol triblock copolymer (M15P10). Unlike polymer solutions composed of methacrylated CS alone or M15P10 alone, mixtures containing CS and M15P10 showed strain-softening, thermo-sensitive and shear-thinning properties. The 3D printing of this hydrogel resulted in the generation of constructs with tailorable porosity and embedded chondrogenic cells remained viable and proliferating over a culture period of 6 days [86] confirming the potential of this hydrogel solution for injectable, cell laden tissue engineering constructs.

While 3D printing and injection molding allow engineered tissues to be structurally modified on the macro scale, they do not offer the nanoscale structural control of electrospinning. As mentioned previously, electrospun, nanofibrous scaffolds have many advantages over monolithic hydrogels for some tissue engineering applications, namely for dermal grafts. They structurally mimic the extracellular matrix, allow for cell adhesion, allow oxygen to permeate, and allow cell waste to escape, making them ideal for wound healing [42]. Unmodified CS and the aforementioned hydrogel systems do not allow for electrospinning as do polycaprolactone-based systems [83]. Using acetic acid and water to reduce the use of potentially toxic organic solvents, Sadeghi, et al. have electrospun a gelatin/polyvinyl alcohol/chondroitin sulfate nanofibrous scaffold for skin tissue engineering [87,88]. Results indicated that the gels were not cytotoxic and L929 fibroblasts attach and proliferate on the scaffolds without issue, as assessed via scanning electron microscopy [87], indicating they may be suitable for skin remodeling and regeneration. Further, this suggests that with further work, viable methods for electrospinning biocompatible scaffolds at scale will be realized.

In summary, Chondroitin sulfate-based gel systems have been developed for cartilage and bone repair, and wound healing due to their ability to direct cell attachment, cell lineage commitments, and differentiation [60,61]. Similar to HA, CS lacks the essential mechanical properties necessary for implantation. The bulk of current research, therefore, focuses on adding CS to bulk scaffolds for mechanical support or crosslinking CS. CS, when self-gelled or crosslinked to another organic or inorganic agent, has been shown to promote mineralization and osteogenesis, chondrogenesis, and wound healing in cell-laden tissue engineering constructs. Collectively, these studies demonstrate the key role CS plays in serving as a depot for growth factors to rapidly make them available as necessary for regeneration and engineering of new tissues. They also highlight the importance of highly charged sulfate groups on CS for binding of these factors and aggregation of ions such as the calcium and phosphate required for skeletal and dental bone mineralization. Many advances have also been made in making these constructs injectable and customizable using 3D printing and newer crosslinking modalities, while reducing the use of harsh, cytotoxic chemicals. A summary of CS hydrogel system and their tested behavior in vitro and in vivo can be found in Table 3.

## 4. Chondroitin Sulfate-Hyaluronic Acid Hybrid Tissue Engineering Systems

More recently, there has been an increase in papers published describing tissue engineering systems that utilize more than one GAG to explore their synergy with respect to directing cell behavior. Fernandes-Cuhna, et al., for example, investigated the ability of an HA+CS construct to support MSCs and accelerate corneal healing in several mouse models of corneal injury. The results showed that a once-daily application of MSCs in HA/CS enhances epithelial cell proliferation and wound healing after injury to the cornea. It also reduced scar formation, neovascularization, and hemorrhage after alkaline corneal burns [89]. Building on single GAG hydrogels, like those formed from methacrylated CS, recent studies show that CS and HA alone can form scaffolds by crosslinking methods including functionalization with tyramine. Tyramine functionalized CS and HA can be covalently bonded to form strong, elastic gels that offer good viability when seeded with MSCs [90]. Similarly, electrospun scaffolds like the gelatin/PVA/CS mentioned earlier can instead be formed using gelatin, HA, and CS. These gels, loaded with sericin, showed several-fold increases in proliferation of human foreskin fibroblast, human keratinocyte and human MSCs, and supported epithelial differentiation in all three cell types. In addition, expression of some dermal proteins was achieved [91]. HA and CS have also been combined with gelatin and silk fibroin for cartilage tissue engineering. This combination was found to induce chondrogenesis of bone marrow MSCs [92]. These experimental systems lend support to the idea that tissue engineering constructs will only improve as the appropriate GAGs for each system are incorporated.

Research using CS or HA alone in tissue engineering has progressed drastically since the inception of tissue engineering in the late 1980s. With this comes the transition to incorporation of both HA and CS into scaffolds to better recapitulate the native extracellular matrix and improve tissue regeneration. Preliminary research combining both suggests they may work to together to improve regeneration of the cornea, articular cartilage, or skin following trauma.

## 5. Dermatan Sulfate

DS is found in the cornea, where it maintains optical clarity, and in the sclera, where it helps to maintain the eye’s overall shape [93]. Further, it is found in blood vessel walls, heart valves, and the umbilical cord during pregnancy where it plays a key role in regulation of the extracellular matrix [93]. Its composition is very similar to that of CS, as demonstrated by its former name CS type B, however I-iduronic acid a C5 epimer of glucuronic acid, substitutes for hexuronic acid found in CS [50]. Sulfation is found on C4 or C6 of the galactosamine ring and sulfation levels increase with age [93].

DS has been implicated in the development of many pathologies, such as cancer metastasis [94], connective tissue diseases [95], and inhibited neuron regeneration [96]. Research focusing on DS in tissue engineering is sparse, with the bulk of research focusing on discovery of its functions and some research focusing on DS, modified DS, and DS proteoglycans as therapeutics or as a targeting mechanism for drug delivery [97,98,99,100,101]. This lack of exploration can likely be attributed to two key factors: the recent reclassification of DS from chondroitin sulfate B, and the extreme complexity of DS synthesis and physiological interactions. DS interactions are based upon the composition and sulfate functionalization patterns of the chain allowing for a high diversity of patterns and potential interactions similar to those seen for CS. For example, xyloside-primed dermatan sulfate from breast carcinoma cells has cytotoxic effects and this behavior is only exhibited by DS of a defined disaccharide composition [102]. This is the first example of cytotoxic effects of dermatan sulfate and highlights the complexity of cell interactions with sulfated GAGs. There has, however, been some research focusing on the use of DS in tissue engineering. DS proteoglycans are key moderators of fibrinogenesis and K.M., et al. have shown that this behavior can be recapitulated in vitro when combining DS with collagen scaffolds. Collagen fibril formation was shown to be dependent on DS concentration, with low concentrations resulting in disorganized fibrils and higher concentrations resulting in more organized, but less dense fibrils [103]. A more unique use for DS in tissue engineering may be in surface modification of implantable devices. DS, when combined with chitosan in a multi-layer coating on polyethylene terephthalate surface show high surface wettability and inhibited biofilm formation, two important factors in implantable devices such as vascular prosthetics [104].

It has also been shown recently that mouse embryonic stem cells undergo neuronal differentiation via activation of signal-regulated kinase 1/2 and human neural stem cells undergo neuronal differentiation and neuronal migration in the presence of DS [105]. This lends some promise to the use of DS to drive stem cell differentiation in neuronal tissue engineering constructs similar to the use of HA and CS as mentioned previously.

## 6. Heparan Sulfate and Heparin

Heparan sulfate’s dominant repeating disaccharide unit is composed of glucuronic acid linked to N-acetylglucosamine [1]. Heparan sulfate is considered the most complex GAG and medical uses of this GAG are currently few and far between. Heparin, on the other hand has seen a myriad of medical applications. It is composed primarily of iduronic acid-N-sulfoglucosamine disaccharide units and is heavily sulfated. Many naturally occurring GAGs display a hybrid structure that blurs the line between HS and heparin. It has been proposed that the name heparin be only applied to GAGs containing more N-sulfate groups than N-acetyl groups. This falls in line with the generally accepted distinction that heparin is more highly sulfated than HS [106].

Pan, et al. have combined CS-chitosan scaffolds with heparin-gelatin microspheres to utilize the growth factor sequestering properties of heparin. These gels were formed from oxidized CS and carboxymethyl chitosan using the schiff’s base reaction similar to the gels mentioned previously. Doping with these microspheres accelerated gelation, slowed weight loss, increased water uptake, and increased the compressive modulus over controls. Adipose-derived stem cells showed good viability as they did with the CS-chitosan gels, but had the added benefit of controlled release of incorporated growth factors such as insulin-like growth factor 1, while gels without heparin-gelatin microbeads exhibited burst release. These gels also showed the same injectability of CS-chitosan only gels for non-invasive tissue engineering therapies [107].

Tissue engineering research using heparan sulfate and, in some cases heparan sulfate mimetics [108], has recently increased as it became clear that HS can be administered to injury sites to support bone healing [109] and angiogenesis [110] and might, therefore, confer benefits to tissue engineering constructs. Lee, et al. recently investigated the binding affinity of a myriad of growth factors including TGF-β1, BMP-2, FGF-2, PDGF-BB, and VEGF165 and found it binds them all but with varying affinities, that may depend on the sulfation pattern and composition of the HS used. Further, in a mouse model of osteochondral defect, HA gels loaded with HS and no growth factors or stem cells showed recovery to normal or near normal as measured using the International Cartilage Regeneration and Joint Preservation Society cartilage injury evaluation scoring system. Gels containing HS were also the only to support regeneration of bone and cartilage, while HA only gels did not support bone regeneration [111]. Sefkow-Werner, et al. also noted that HS as part of a gel construct including bone morphogenic protein 2 and cyclic RGD worked synergistically with the growth factor and cell adhesion molecule in eliciting osteogenic differentiation and promoting enhanced and sustained signaling [112]. Possibly more importantly, they developed a streptavidin-based system that allows for tunable amounts of each ligand to be immobilized in a gel to investigate how their relative densities affect cell behavior with the potential to further the use of GAGs to improve tissue engineering constructs. While work with HS and heparin in tissue engineering is nascent with the exception of controlled release of growth factors, as more is learned about these important extracellular matrix components we anticipate that, as with other GAGs, their uses will increase.

## 7. Keratan Sulfate

Keratan sulfate is the exception to the usual hexuronic acid plus amino sugar composition of GAGs and is instead composed of galactose and acetylated glucosamine [1]. KS is a widely distributed GAG, even more so than those previously mentioned. It is found in the weight bearing connective tissues and epithelial tissues, as well as in the central and peripheral nervous systems, where it plays a key role in control of ion fluxes between neurons [4]. Cells’ ability to respond to biochemical stimuli is contingent on the ability to control and sense ion fluxes and KS plays a key role in the regulation of this, and further, the pathophysiology neuronal disorders such as epilepsy [4]. Control of charges and ion gradients such as these also play a role in adhesion, proliferation, and differentiation of cells, and even wound healing [113]. This importance in chemical and ionic signaling is further highlighted by the fact that the brain is the second most KS rich organ following the eyes, where, as part of a KS proteoglycan, it plays a role in neurogenesis, demarcation of brain areas, direction of neuronal growth, and repair processes [5].

Despite its ubiquity, Keratan sulfate’s interactions and uses in tissue engineering are the least understood out of all of the GAGs [114] and its applications in tissue engineering to date are nonexistent. The majority of current research focuses on discovery of KS’ role in regenerative neural processes [114], airway/lung inflammation [115,116], and infection [117], emphysema [118], corneal dystrophy [119], cancer malignancy [120], and specialized functions in other species [121,122]. An emphasis has also been placed on analyzing sources for isolatable KS [123], and KS as a contaminant in CS purification [124,125,126].

## 8. Summary and Future Directions

**Figure 3:**
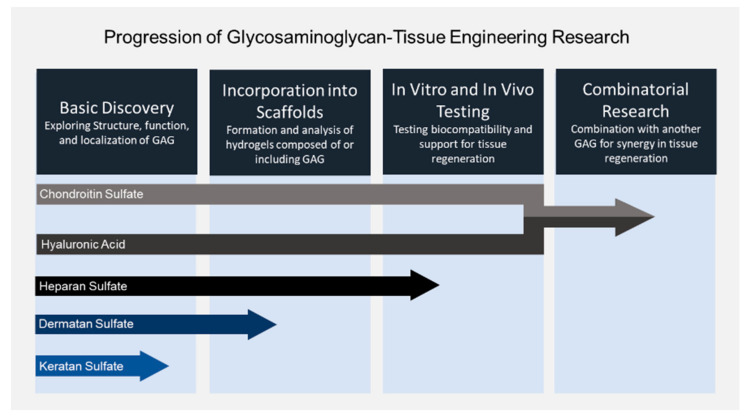
The four generalized steps of incorporating glycosaminoglycans into tissue engineering and the relative progress of each in this process.

GAGs are used in tissue engineering constructs to recapitulate the ECM and, thereby, drive stem cell differentiation or retention of phenotype of implanted cells. This allows them to be used as implants for regeneration of damaged tissue. Recent research in this field focuses on tissue engineering constructs for wound healing in skin and cornea, restoring damaged cartilage, such as articular cartilage and the NP, restoring bone, and neuronal regeneration. Research in the last five years generally focuses on three key areas: (1) overcoming the physical limitations of GAGs alone, by developing scaffolds that mimic the rheological properties of native tissues that can be doped with CS to present its moieties; (2) exploration of growth factor and ion sequestering by GAGs in TECs and how this affects their ability to promote cell differentiation and tissue regeneration with one or multiple GAGs; (3) advancement of hydrogel crosslinking technologies to reduce cytotoxicity of components and reagents and confer new, useful properties, such as sheer thinning/thermos-responsiveness for 3D printing, or to allow for GAG-only scaffolds that do not require polymers such as chitosan, collagen, PEGDA, etc. Area (1) has been investigated quite heavily to date, but new potential applications are still emerging, and (2) and (3) leave much room for exploration. We have seen that incorporation of gel formations with one GAG to drive cell fate and tissue regeneration has been heavily explored in some tissues, such as cartilage and bone with some emerging research in combining multiple GAGs into a construct. Moreover, each GAG has been investigated to a different extent. The general trends in tissue engineering using GAGs and where each GAG stands in the process are summarized in Figure 3. Moving forward, we expect to see more tissue engineering constructs that incorporate multiple GAGs to elucidate their synergistic effects on stem cell fate and their composite potential for tissue regeneration, whether the form is as functionalized, crosslinked GAGs alone or GAGs immobilized or crosslinked into a gel composed of another polymer.

All tissues contain a complex mix of different GAGs with different compositions, as described previously. It follows, then, that future research will increasingly focus on different “versions” of the same GAG and combinations of different GAGs in different ratios to recapitulate native tissue. This will be especially true as the wealth of knowledge regarding each GAG individually grows. Moving forward, we expect to see an increasing number of tissue engineering constructs with two or more GAGs and insight into their interplay. We also expect increased application of GAG based or GAG+ polymer gels in a wider variety of biological systems.

## Figures and Tables

**Table 1:** The hexuronic acid and amino sugar constituents of each glycosaminoglycan.

Glycosaminoglycan	Hexuronic Acid	Hexosamine
Chondroitin Sulfate [1]	glucuronic Acid	N-acetylgalactosamine
Dermatan Sulfate [1]	Iduronic Acid	N-acetylgalactosamine
Keratan Sulfate [1]	galactose	N-acetylglucosamine
Heparan Sulfate [1]	glucuronic Acid	N-acetylglucosamine
Hyaluronic Acid [1]	glucuronic Acid Unsulfated	N-acetyl-D-glucosamine Unsulfated

**Table 2:** Summary of all aforementioned hyaluronic acid hydrogel types and their tested behavior in vitro and in vivo.

HA Type	Copolymer Type	Biological Testing	Biological Outcome
Unmodified HA^46^	Epoxy-Agarose	Seeded RAW 264 macrophages Seeded 3T3 fibroblasts Mouse model of full thickness dermal wound	Increased TNF-α secretion Upregulated collagen I and III Accelerated healing in vivo
Unmodified HA^40^	Polycaprolactone	Seeded human skin keratinocytes and fibroblasts + epidermal growth factor Mouse model of full thickness wound	Upregulated collagen I and III and TGF-ß Accelerated healing in vivo
Methacrylated HA^22^		Seeded Human Mesenchymal Stem Cells	Osteogenic differentiation
Tyramine-HA^43^	Silk Fibroin	Seeded Human Mesenchymal Stem Cells	Supports Adhesion and Proliferation
HA-tyramine^37^	Gelatin-Tyramine	Seeded with C2C12 myoblasts Spin coated membranes seeded with A549 alveolar epithelial cell^34^	Induced myotubule formation Supported epithelial differentiation in the presence of growth factors
HA + Dithiol linker peptide^26^		Seeded Human Dermal Fibroblasts	Supports Cell adhesion and proliferation
HA + catecholamine^16^		Human adipose derived stem cells Human hepatocytes Implantation in athymic mice	Supports Cell Adhesion and proliferation of both stem cells and hepatocytes Implanted hepatocytes secreted albumin detectable in the blood stream in vivo
Esterified HA^23^		Bone Marrow Concentrate	Osteogenic differentiation
HA-hydrazine^17^	HA-aldehyde, HA-aldehyde, and/or collagen	Seeded with human Mesenchymal stem cells Doped with cellulose nanocrystals and seeded with 3T3 fibroblasts^17^	Supports Cell adhesion and proliferation Increased stiffness and retained support of cell viability
HA-monoaldehyde^33^	Carbohydrazide gelatin	Seeded with human umbilical cord endothelial cells Rat aortic ring assay	No toxicity Endothelial migration and microvascular extension
Electrospun HA^39^	Electrospun gelatin	Mouse model of thermal burns	Improved burn wound healing

**Table 3:** Summary of all aforementioned chondroitin sulfate hydrogel types and their tested behavior in vitro and in vivo.

CS Type	Copolymer Type	Biological Testing	Biological Outcome
Unmodified CS^51^	Collagen	Seeded human mesenchymal stem cells and blood mononuclear cells together Implantation under mouse dorsal skin	Bolstered ability of mesenchymal stem cells to reduce inflammation in blood mononuclear cells Chondrogenic differentiation Low neutrophil infiltration in vivo
Unmodified CS^53^	Collagen II + Genipin	Seeded human adipose derived stem cells Rat model of nucleus pulposus degeneration	Chondrogenic differentiation (nucleus pulposus specific) Regeneration of nucleus pulposus in vivo
CS + chitosan nanoparticle^79^	chitosan–Poly(hydroxybutyrate-co-valerate)	Seeded human adipose derived stem cells Rat model of nucleus pulposus degeneration	Chondrogenic differentiation Regeneration of nucleus pulposus in vivo
Unmodified CS^52^	Chitosan + SHC* + BGP*	Seeded with L929 Fibroblasts	Supports Cell adhesion and proliferation
Unmodified CS^54^	Hydroxy–Butyl–Chitosan	Seeded with Human adipose derived stem cells	Supports Cell adhesion and proliferation
Unmodified CS^68^	Polyethylene glycol + CS binding peptide + crosslinker peptide	Embryonic Chick Dorsal Root Ganglia	Enhanced nerve growth
Methacrylated CS^58^		Seeded with rate central nervous system neurospheres Critically sized femoral defect in rats	Promotes survival and self-renewal of neurospheress Bone regeneration in constructs containing BMP-2
Methacrylated CS^68^	Polyethylene glycol	Seeded with human mesenchymal stem cells Mouse model of calvarial defect	CS-dependent calcium and phosphate sequestration Osteogenic differentiation and mineral deposition Bone regeneration
Unmodified CS^69^	Alginate	Seeded rat bone marrow cells Implantation in rat dorsal subcutis	CS-dependent Osteocalcin deposition CS-dependent Osteogenesis in vitro
CS + Tyramine^75^	Hydroxymethyl Pullulan	Seeded with porcine articular chondrocytes Subcutaneous implantation in mice	Supports Cell adhesion and proliferation Cartilaginous matrix deposition Good biocompatibility in vivo
Oxidized CS^76^	Pullulan-adipic hydrazide	Seeded rabbit articular chondrocytes	Supports chondrogenesis
Unmodified CS^81^	Polyvinyl alcohol and gelatin	Seeded with L929 fibroblasts	Supports Cell adhesion and proliferation
Methacrylated CS^59^	pHPMAlac-PEG triblock polymer*	Seeded with Chondrogenic ATDC5 cells	Supports cell survival and proliferation
CS-Graphene Oxide^72^		Seeded with human mesenchymal stem cells	Supports cell proliferation and deposition of collagen matrix
Unmodified CS^80^	PDMAEA-Q*	Tested Adhesion to porcine skin in vitro Seeded with HEPG2 human liver cancer cells	Strong adhesion to tissue Supports cell survival and proliferation

## Data Availability

No new data was created or analyzed in this study. Data sharing is not applicable in this article.
